# Down-regulation of Long Non-coding RNA LINC01554 in Hepatocellular Cancer and its Clinical Significance

**DOI:** 10.7150/jca.40512

**Published:** 2020-03-05

**Authors:** Yuan Ding, Zhongquan Sun, Sitong Zhang, Yining Chen, Bo Zhou, Guogang Li, Qiang Sun, Dongkai Zhou, Yao Ge, Sheng Yan, Weilin Wang

**Affiliations:** 1 Department of Hepatobiliary and Pancreatic Surgery, The Second Affiliated Hospital, Zhejiang University School of Medicine, Hangzhou 310009, China; 2 Key Laboratory of Precision Diagnosis and Treatment for Hepatobiliary and Pancreatic Tumor of Zhejiang Province, Hangzhou 310009, China; 3 Research Center of Diagnosis and Treatment Technology for Hepatocellular Carcinoma of Zhejiang Province, Hangzhou 310009, China; 4 Clinical Medicine Innovation Center of Precision Diagnosis and Treatment for Hepatobiliary and Pancreatic Disease of Zhejiang University, Hangzhou 310009, China; 5 Clinical Research Center of Hepatobiliary and Pancreatic Diseases of Zhejiang Province, Hangzhou 310009, China

**Keywords:** long non-coding RNA, LINC01554, hepatocellular cancer, accurate diagnosis, prognostic prediction

## Abstract

**Background**: For high morbidity rate but lack of early accurate screening, hepatocellular cancer (HCC) manifests as the fourth leading cause of cancer related death worldwide. Accumulating evidence demonstrated that a series of long noncoding RNA (lncRNA) have strong association with pathogenesis and clinical evaluation of HCC. LINC01554, one kind of lncRNA, has been found specifically enriched in liver tissue. However, the relationship between LINC01554 expression and HCC tumorigenesis remains unclear.

**Methods**: The relative LINC01554 expression was measured in HCC tissues of 138 patients and several HCC cell lines using quantitative real-time PCR. Patients were grouped according to individual LINC01554 expression. Then, the potential association between LINC01554 expression in HCC tissues and clinical characteristics as well as prognostic information of patients was evaluated.

**Results**: Compared to correspongding adjacent liver tissues, the LINC01554 expression in HCC was significantly down-regulated (*P*=0.001). And its expression levels in HCC cell lines were also remarkably lower than that in normal human hepatocyte cell line (*P*<0.001). Besides, the expression level of LINC01554 was significantly related to tumor size, multiple lesions, TNM stages, tumor recurrence rate as well as long-term survival in HCC patients (*P*<0.05).

**Conclusion**: The research revealed that LINC01554 was down-regulated in HCC and it could be used for the accurate diagnosis and prognostic prediction of HCC patients.

## Introduction

On account of high malignancy but limited valid early screening methods, hepatocellular cancer (HCC) remains as the fourth leading cause of cancer related death in the past 20 years.^1^ Although surgery and drug therapy have being used in clinical treatment, the long-term survival rate of HCC patients is still unsatisfactory.^2^,^3^ In clinical practice, there lacks appropriate accurate diagnostic and prognostic marker for detecting HCC.^4^ Even as the most common tumor marker used in liver diseases, the serum alpha-fetoprotein (AFP) value has been excluded from HCC clinical guidelines by both American and European association of liver disease, which results from its proved unqualified sensitivity and specificity.^5^,^6^ Therefore, it attaches great importance to identifying some reliable early-warning biomarkers for accurate HCC diagnosis and prediction of patient's long term outcome. Recently, accumulating researches have reported that long noncoding RNA (lncRNA) expression has strong association with occurrence and progress of HCC.^7^

The lncRNA is a special type of RNA molecule longer than 200 nucleotides. Despite that lncRNA is not translated into proteins, it has been elucidated to function as critical regulatory roles in diverse cellular process.^8^ With efforts made to reveal different functional mechanisms of lncRNAs, nowadays emerging evidences elucidate that lncRNAs make critical influence on the biological regulations in terms of genetic transcription, protein synthesis, cell proliferation as well as tumorigenesis.^9^,^10^ Based on these, lncRNAs can be transformed as diagnostic, prognostic and targeted therapeutic biomarkers for clinical practice. For instance, growth arrest-specific transcript 5 (GAS5), an up-regulated lncRNA in HCC, has been proved to display anti-apoptosis effect on the tumor progression.^11^ Moreover, Li reported what the over-expressed lncRNA highly up-regulated in liver cancer (HULC) manifests evident relationships with process of tumorigenesis in HCC.^12^ Patients with high HULC expression levels presented with significant increased risk in HCC recurrence and poor survival.^13^ Besides, a series of lncRNAs, such as metabolism-induced tumor activator 1 (MITA1),^14^ Linc-GALH,^15^ and KTN1 antisense RNA 1 (KTN1-AS1),^16^ also have been proved to be effective in accurate diagnosis as well as prognostic prediction of HCC.

Long intergenic non-protein coding RNA 1554 (LINC01554), one kind of lncRNA, has been sequenced to locate at chromosome 5 in human tissues.^17^ There are some researches to explore the potential association between LINC01554 expression and disease development. Ryaboshapkina M reported that decreasing levels of LINC01554 expression have strong association with advancing liver fibrosis for NAFLD patients.^18^ In Fan's study, LINC01554 combined with other lncRNAs can function as a novel model to provide more powerful prognostic information beyond conventional clinicopathological factors in esophageal cancer patients.^19^ However, the research about revealing the clinical and prognostic significance of LINC01554 expression in HCC remains blanked.

In present study, we aimed to evaluate the relative expression of LINC01554 in HCC. Then, we investigated the accurate diagnostic and prognostic predicting abilities of LINC01554 for HCC patients.

## Materials and Methods

### Patient data and specimens

In total, 138 inpatients undergoing hepatectomy at the First Affiliated Hospital, School of Medicine, Zhejiang University from January 2013 to August 2014 were included into the present study. With use of hospital medical database system, the perioperative data of patients were collected, including age, gender, hepatitis B virus infection, liver fibrosis, liver function indicators, tumor states, TNM stages and so on. After hepatectomies, the liver lesions of patients were sent to the histological examinations, and the final reports were reviewed by the identical experienced pathology doctor.

All patients were diagnosed with HCC according to the histological examination of liver lesions. The HCC specimens and corresponding adjacent tissues of them were obtained from surgical resections and all isolated samples preserved in liquid nitrogen were transported to laboratory within 30 min. Corresponding adjacent tissues were harvested 3cm from the edges of the tumor lesion. Valid survival data was obtained over a period of 60 months in 85 patients and tumor recurrence information was available in 102 patients.

### Cell culture

The human normal hepatocyte cell line QSG-7701 and several human HCC cell lines (PLC, Hep3B, Huh7, SMMC-7721) were cultured in DMEM (Gibco, USA) containing heat-inactivated 10% fetal bovine serum (FBS, Gibco, USA), 100mg/mL streptomycin and 100U/mL penicillin in a humidified atmosphere of 5% CO_2_ at 37℃.

### RNA extraction and cDNA synthesis

Total RNA was extracted from cells, tumor and adjacent tissues using the Trizol reagent (Invitrogen, Carlsbad, CA, USA). The concentration of RNA was measured by Nanodrop 2000 spectrophotometer (Thermo Scientific Inc., Waltham, MA, 93 USA). The RNA was reversely transcribed into cDNA using HiScript II Reverse Transcriptase SuperMix with gDNA wiper (Vazyme, Nanjing, China) according to the manufacturer's instructions.

### Real-time PCR analysis

Quantitative real-time polymerase chain reaction (RT-PCR) was implemented to determine the expression level of LINC01554 using Fast Start Universal SYBR Green Master ROX (Roche, Basel, Switzerland) following the manufacturer's instructions. The sequences of all used PCR primers were as follows (5'-3'): LINC01554: GGAGGTCGGTTGATGAGCAGT (forward), GTCAAGCCTGTGTGTCATCGTT (reverse); GAPDH: CAGGAGGCATTGCTGATGAT (forward), GAAGGCTGGGGCTCATTT (reverse). Combined with Glyceraldehyde-3-phosphate dehydrogenase (GAPDH) expression as the endogenous control, relative LINC01554 expression was calculated by the comparative Ct method formula 2^-ΔΔCt^. Every samples was tested in triplicates.

### Statistical analysis

Generally, the continuous variables were expressed as mean value with standard deviation or number (percentage). Statistical software package SPSS19.0 (SPSS Inc, Chicago, IL, USA) was used to accomplish all statistical analysis. Quantitative variables were assessed by Student's t test, while Chi-square test and Fisher's exact test were applied to analyze qualitative and categorical data. Overall survival curves were plotted with use of the Kaplan-Meier method. All statistical differences of *P*<0.05 were considered statistically significant.

### Ethical statement

The study was in accordance with the ethical guidelines of the 1975 Declaration of Helsinki. All participants had signed written informed consent. Ethical approval was obtained from the Ethics Committee of the First Affiliated Hospital, School of Medicine, Zhejiang University. All data were analyzed anonymously and identified prior to analysis.

## Results

### LINC01554 was down-regulated in HCC tissues

**Figure 1:**
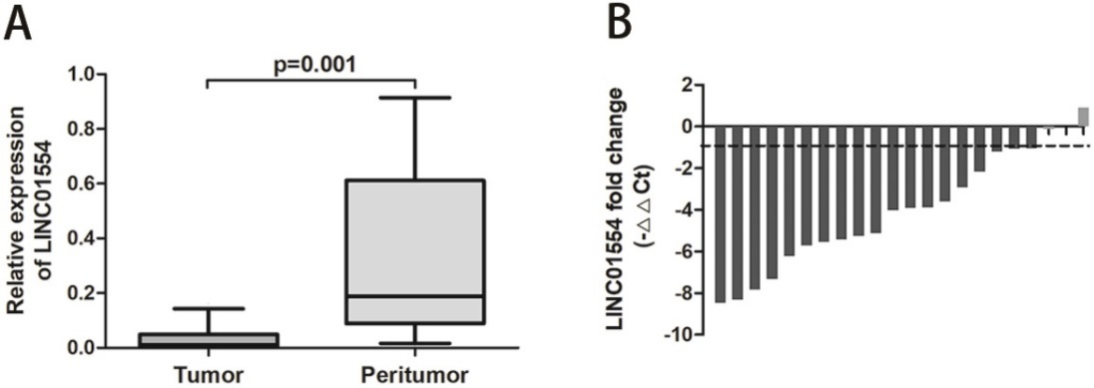
Relative expression of LINC01554 in clinical HCC and adjacent liver tissues. **(A)** LINC01554 expression was significantly lower in HCC tissues than that in adjacent liver tissues (*P*=0.001). **(B)** Waterfall plot showed that LINC01554 was down-regulated by at least twofold in 86.3% of paired HCC tissues.

To explore the potential function of LINC01554 in HCC, we detected the LINC01554 expression in paired clinical HCC and adjacent liver tissues using real-time PCR. As showed below, LINC01554 expression was significantly down-regulated in HCC tissues compared to matched adjacent liver tissues (*P*=0.001; Figure 1A). Moreover, waterfall plot showed that LINC01554 was down-regulated by at least twofold in 86.3% of the HCC tissues. (Figure 1B).

### The down-regulation of LINC01554 in HCC cell lines

**Figure 2:**
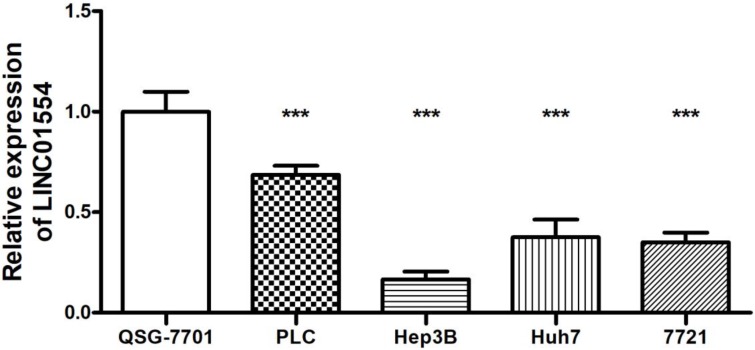
Analysis of LINC01554 expression in HCC and normal liver cell lines. Compared to normal liver cell line QSG-7701, LINC01554 was significantly down-expressed in several HCC cell lines (PLC, Hep3B, Huh7, SMMC-7721). ***P<0.001.

To further evaluate the association between LINC01554 and HCC, the expression of LINC01554 was measured in human normal hepatocyte cell line QSG-7701 as well as various HCC cell lines (PLC, Hep3B, Huh7, SMMC-7721). As shown in Figure 2, LINC01554 was also found to be significantly down-expressed in HCC cell lines compared to normal liver cell lines. Based on the above, the verification results of both tissues and cell lines implied that LINC01554 functioned as a tumor suppressor in the progress of HCC.

### Correlation of LINC01554 and clinical characteristics in HCC patients

According to the values of LINC01554 expression, 138 HCC patients were cataloged into high or low expression groups. Among these HCC patients, there were 117 males (84.7%) and 21 females (15.3%) with mean age of 54.95 years old (range from 26 to 81 years old). About 119 (86.2%) of them were complicated with hepatitis B and 82 (59.4%) presented with the basic medical history of liver cirrhosis. The clinical characteristics of HCC patients were listed in Table 1.

By comparing the patients in high and low LINC01554 expression groups, we found that patients in low LINC01554 expression group exhibited significantly higher incidence of huge (tumor diameter >5cm) and multiple lesions than that in high expression group (*P*<0.05, Table 1). Besides, the HCC patients with lower LINC01554 expression level had more severe status in classification of TNM stages (P=0.010) and higher risk of tumor recurrence (*P<*0.05, Table 1). However, there were no significant associations in characteristics of age, gender, hepatitis B infection, liver cirrhosis, AFP, ALT, total bilirubin, microvascular invasion and differentiation. These suggested that LINC01554 expression was related to occurrence and development of HCC.

### Low LINC01554 expression level is associated with poor survival in HCC patients

**Figure 3:**
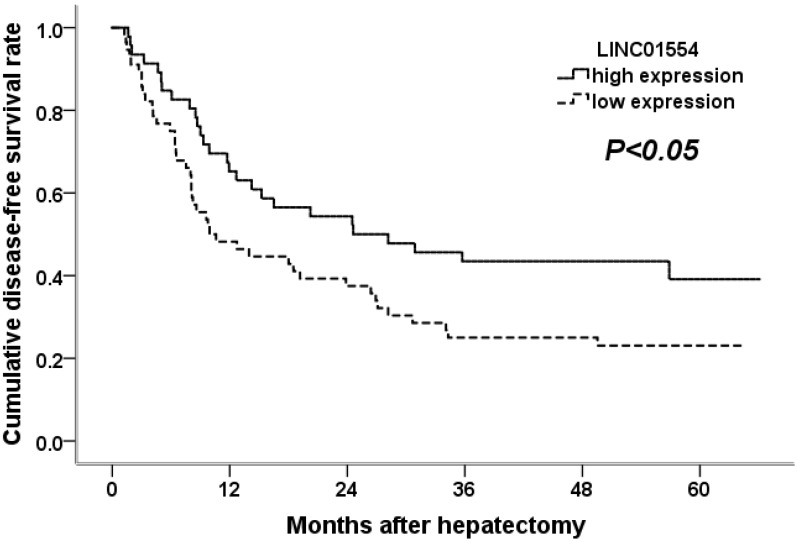
Cumulative disease-free survival curves of patients in high and low LINC01554 expression groups.

**Figure 4:**
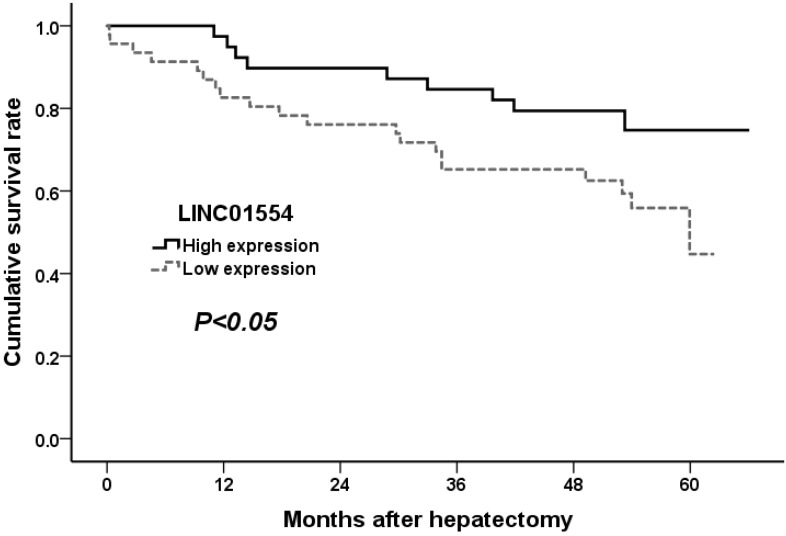
Cumulative survival curves of patients in high and low LINC01554 expression groups.

To confirm the potential ability of LINC01554 in predicting patient survival, we performed Kaplan-Meier analysis in aspects of postoperative tumor recurrence as well as long term survival. As shown in Figure 3, HCC patients with lower LINC01554 expression presented with shorter tumor-free survival time and higher tumor recurrence rates (*P*<0.05). What' more, the evaluation of long term survival demonstrated that low LINC01554 expression level was significantly associated with poor outcomes in HCC patients (*P*<0.05, Figure 4).

## Discussions

In the present study, we for the first time performed the independent research on relationship between LINC01554 expression and liver cancer. We found that LINC01554 was down-regulated in HCC tissues comparing to corresponding adjacent liver tissues, which was consistent with following validation in HCC lines. What's more, the down- regulation of LINC01554 was proved to be related to the severity classification, postoperative recurrence as well as long-term survival of HCC patients. Thus, it indicated that LINC01554 functioned as a tumor suppressor in the progress of HCC.

For high morbidity rate but lack of early accurate screening methods, HCC manifests as the fourth- cause of caner related death over the world.^20^ Although plenty of biomarkers had been evaluated for early diagnosis, condition assessment and survival prediction for HCC patients, ideal indicators with high accuracy and sensitivity remained to be explored.^21^ Moreover, the target therapy for treating advanced HCC remained limited for lack of definite abnormal gene targets.

Since being proposed firstly in 1991,^22^ lncRNAs have been proved to fulfill vital functions in the regulation of gene expression in accumulating researches. Therefore, the dysregulation of some specific lncRNAs can make influence on various abnormal intracellular regulatory process, such as cancer initiation and progression.^23^ For example, growth arrest-specific transcript 5 (GAS5), a down-expressed tumor-suppressor lncRNA in gastric cancer, is identified to enhance various tumorigenesis by decreasing YBX1-transactivated p21 expression, 24 negatively regulating miR-222 expression and so on.^25^ As for liver tumors, the lncRNA down-regulated in liver cancer stem cells (DILC) can markedly facilitate HCC initiation and progression via IL-6/STAT3 axis,^26^ while highly expressed LINC00210 can significantly drive propagation of liver tumor initiating cells through activating Wnt/β-catenin signaling.^27^ In addition, more and more researches have explored the potential diagnostic as well as prognostic abilities of lncRNAs in a variety of diseases. In Zhou's study, they succeeded in identifying and establishing a four-lncRNA-focus risk model for screening as well as predicting overall survival of patients with multiple myeloma, which had great potential clinical value for application.^28^ On account of the varied survival periods in different HCC patients, defining the distinct prognostic risk levels with lncRNAs can help optimize the ideal option of personalized therapy.

According to the data in GTEx database, LINC01554 enrichment is specifically found in liver, salivary gland and kidney. With use of genome and transcriptome sequencing technologies, the entire sequence of LINC01554 has been identified to be encoded at chromosome 5q15.^29^ There are some researches indicating that chromosomal locus 5q15 is directly associated with several novel hepatic liver function related receptors. In Gaieb's study, they proved that 5q15 is the specific ligand binding to the nuclear receptor farnesoid X receptor (FXR).^30^ As reported, FXR can make novel regulatory influence on metabolic homeostasis of nutrients, fats, sugars and proteins by bile acids in liver cells.^31^ Meanwhile, it has been approved that FXR can functionally prevent liver cancer by protecting tumor suppressor proteins from degradation via C/EBPβ-HDAC1 complexes. 32 Thus, dysregulation of LINC01554 possibly leads to abnormal FXR expression in liver cells, followed by activating proliferative potential and weakening intracellular tumor suppressing regulation. To validate the exact mechanisms of LINC01554 in human HCC, more large prospective researches are needed in the future.

In conclusion, the lncRNA LINC01554 was universally down-expressed in HCC, and it had significant associations with tumor size, multiple lesions and TNM stages of patients. As for postoperative fellow-up, HCC patients with low LINC01554 expression generally presented with higher tumor recurrence rate and shorter tumor-free survival time. Therefore, LINC01554 expression can be used to provide clinical doctors with accurate assessment as well as prognostic prediction of HCC patients.

## Figures and Tables

**Table 1:** Correlation of LINC01554 and clinical characteristics in HCC patients

Characteristics	Total	LINC01554 expression		*P* value
		High (n=69)	Low (n=69)	
**Age, ys**	138	55.96±11.89	53.93±10.69	0.294
**Gender**				0.477
Male	117	57 (0.83)	60 (0.87)	
Female	21	12 (0.17)	9 (0.13)	
**Hepatitis B**				0.805
Positive	119	59 (0.86)	60 (0.87)	
Negative	19	10 (0.14)	9 (0.13)	
**Cirrhosis**				0.729
Positive	82	42 (0.61)	40 (0.58)	
Negative	56	27 (0.39)	29 (0.42)	
**ALT (U/L)**	138	40.11±55.98	31.97±20.09	0.257
**Total bilirubin (μmol/L)**	138	18.88±33.80	12.52±4.89	0.124
**AFP (ng/l)**				0.160
>400	52	22 (0.32)	30 (0.44)	
≤400	86	47 (0.68)	39 (0.56)	
**Tumor diameter**				0.046*
>5cm	93	41 (0.59)	52 (0.75)	
≤5cm	45	28 (0.41)	17 (0.25)	
**Multiple lesions**				0.029*
Positive	26	8 (0.12)	18 (0.26)	
Negative	112	61 (0.88)	51 (0.74)	
**Microvascular invasion**				0.094
Positive	41	16 (0.23)	25 (0.36)	
Negative	94	50 (0.77)	44 (0.64)	
**Differentiation**				0.233
High/Moderate	73	40 (0.58)	33 (0.48)	
Low	65	29 (0.42)	36 (0.52)	
**TNM stage**				0.010*
I~II	96	55 (0.80)	41 (0.59)	
III~IV	42	14 (0.20)	28 (0.41)	
**Tumor recurrence**				0.031*
Positive	70	29 (0.63)	41 (0.73)	
Negative	32	17 (0.37)	15 (0.27)	

ALT= alanine aminotransferase; AFP= alpha fetal protein; TNM= tumor-node-metastasis; *P<0.05; Values are mean ± standard deviation or n (%).

## Abbreviations

HCC: hepatocellular cancer (HCC)
lncRNA: long noncoding RNA
AFP: alpha-fetoprotein
GAS5: growth arrest-specific transcript 5
HULC: highly up-regulated in liver cancer
MITA1: metabolism-induced tumor activator 1
KTN1-AS1: KTN1 antisense RNA 1
LINC01554: long intergenic non-protein coding RNA 1554
RT-PCR: real-time polymerase chain reaction
GAPDH: glyceraldehyde-3-phosphate dehydrogenase
DILC: down-regulated in liver cancer stem cells
FXR: farnesoid X receptor

## References

[1] Bray F, Ferlay J, Soerjomataram I (2018). Global cancer statistics 2018: GLOBOCAN estimates of incidence and mortality worldwide for 36 cancers in 185 countries. CA Cancer J Clin.

[2] Wallace MC, Preen D, Jeffrey GP (2015). The evolving epidemiology of hepatocellular carcinoma: a global perspective. Expert Rev Gastroenterol Hepatol.

[3] van den Bosch MA, Defreyne L (2012). Hepatocellular carcinoma. Lancet.

[4] Ferrín G, Aguilar-Melero P, Rodríguez-Perálvarez M (2015). Biomarkers for hepatocellular carcinoma: diagnostic and therapeutic utility. Hepat Med.

[5] Bruix J, Sherman M (2011). American Association for the Study of Liver Disease. Management of hepatocellular carcinoma: an update. Hepatology.

[6] European Association for the Study of the Liver, European Organisation for Research Treatment of Cancer (2012). EASL-EORTC clinical practice guidelines: management of hepatocellular carcinoma. J Hepatol.

[7] Huo X, Han S, Wu G (2017). Dysregulated long noncoding RNAs (lncRNAs) in hepatocellular carcinoma: implications for tumorigenesis, disease progression, and liver cancer stem cells. Mol Cancer.

[8] Shi X, Sun M, Liu H (2013). Long non-coding RNAs: a new frontier in the study of human diseases. Cancer Lett.

[9] Bonasio R, Shiekhattar R (2014). Regulation of transcription by long noncoding RNAs. Annu Rev Genet.

[10] Sanchez Calle A, Kawamura Y, Yamamoto Y (2018). Emerging roles of long non-coding RNA in cancer. Cancer Sci.

[11] Tao R, Hu S, Wang S, er al (2015). Association between indel polymorphism in the promoter region of lncRNA GAS5 and the risk of hepatocellular carcinoma. Carcinogenesis.

[12] Tao R, Hu S, Wang S (2015). Association between indel polymorphism in the promoter region of lncRNA GAS5 and the risk of hepatocellular carcinoma. Carcinogenesis.

[13] Panzitt K, Tschernatsch MM, Guelly C (2007). Characterization of HULC, a novel gene with striking up-regulation in hepatocellular carcinoma, as noncoding RNA. Gastroenterology.

[14] Ma M, Xu H, Liu G (2019). Metabolism-induced tumor activator 1 (MITA1), an Energy Stress-Inducible Long Noncoding RNA, Promotes Hepatocellular Carcinoma Metastasis. Hepatology.

[15] Xu X, Lou Y, Tang J (2019). The long non-coding RNA Linc-GALH promotes hepatocellular carcinoma metastasis via epigenetically regulating Gankyrin. Cell Death Dis.

[16] Zhang L, Wang L, Wang Y (2019). LncRNA KTN1-AS1 promotes tumor growth of hepatocellular carcinoma by targeting miR-23c/ERBB2IP axis. Biomed Pharmacother.

[17] Ota T, Suzuki Y, Nishikawa T (2004). Complete sequencing and characterization of 21,243 full-length human cDNAs. Nat Genet.

[18] Ryaboshapkina M, Hammar M (2017). Human hepatic gene expression signature of non-alcoholic fatty liver disease progression, a meta-analysis. Sci Rep.

[19] Fan Q, Liu B (2016). Identification of a RNA-Seq Based 8-Long Non-Coding RNA Signature Predicting Survival in Esophageal Cancer. Med Sci Monit.

[20] Asrani SK, Devarbhavi H, Eaton J (2019). Burden of liver diseases in the world. J. Hepatol.

[21] Sun JH, Luo Q, Liu LL (2016). Liver cancer stem cell markers: Progression and therapeutic implications. World J. Gastroenterol.

[22] Borsani G, Tonlorenzi R, Simmler MC (1991). Characterization of a murine gene expressed from the inactive X chromosome. Nature.

[23] Shi XF, Sun M, Liu HB (2013). Long non-coding RNAs: A new frontier in the study of human diseases. Cancer Lett.

[24] Liu Y, Zhao J, Zhang W (2015). lncRNA GAS5 enhances G1 cell cycle arrest via binding to YBX1 to regulate p21 expression in stomach cancer. Sci Rep.

[25] Li Y, Gu J, Lu H (2017). The GAS5/miR-222 Axis Regulates Proliferation of Gastric Cancer Cells Through the PTEN/Akt/mTOR Pathway. Dig Dis Sci.

[26] Wang X, Sun W, Shen W (2016). Long non-coding RNA DILC regulates liver cancer stem cells via IL-6/STAT3 axis. J Hepatol.

[27] Fu X, Zhu X, Qin F (2018). Linc00210 drives Wnt/β-catenin signaling activation and liver tumor progression through CTNNBIP1-dependent manner. Mol Cancer.

[28] Zhou M, Zhao HQ, Wang ZZ (2015). Identification and validation of potential prognostic lncRNA biomarkers for predicting survival in patients with multiple myeloma. J Exp Clin Cancer Res.

[29] Strausberg RL, Feingold EA, Grouse LH (2002). Generation and initial analysis of more than 15,000 full-length human and mouse cDNA sequences. Proc Natl Acad Sci U S A.

[30] Gaieb Z, Liu S, Gathiaka S (2018). D3R Grand Challenge 2: blind prediction of protein-ligand poses, affinity rankings, and relative binding free energies. J Comput Aided Mol Des.

[31] Massafra V, van Mil SWC (2018). Farnesoid X receptor: A "homeostat" for hepatic nutrient metabolism. Biochim Biophys Acta Mol Basis Dis.

[32] Jiang Y, Iakova P, Jin J (2013). Farnesoid X receptor inhibits gankyrin in mouse livers and prevents development of liver cancer. Hepatology.

